# Minimally Invasive Surgical Repair of a Partial Atrioventricular Canal Defect in a 20-Year-Old Patient—A Case Report and Review of Literature

**DOI:** 10.3390/jcdd9100352

**Published:** 2022-10-14

**Authors:** Horațiu Moldovan, Cristian Bulescu, Mihai Cacoveanu, Cristian Voica, Sabina Safta, Mihai Goicea, Irina Dobra, Iulian Antoniac, Daniela Gheorghiță, Ondin Zaharia

**Affiliations:** 1 Faculty of Medicine, Carol Davila University of Medicine and Pharmacy, 050474 Bucharest, Romania; 2 Department of Cardiovascular Surgery, Emergency Clinical Hospital Bucharest, 014461 Bucharest, Romania; 3 Academy of Romanian Scientists, 54, Spl. Independentei, 050711 Bucharest, Romania; 4 Department of Cardiovascular Surgery, Grigore Alexandrescu Emergency Hospital for Children, 011743 Bucharest, Romania; 5 Faculty of Materials Science and Engineering, Politehnica University of Bucharest, 060042 Bucharest, Romania; 6 Department of Internal Medicine, Prof. Dr. Theodor Burghele Clinical Hospital, 050659 Bucharest, Romania

**Keywords:** atrioventricular defect, mitral valve, minimally invasive surgery, valvular regurgitation

## Abstract

The association of an ostium primum-type defect with a cleft anterior mitral valve is known in the medical literature as the partial form of an atrioventricular canal. We present a case report about a 20-year-old woman with minimal symptomatology that discovered her pathology on routine echocardiography. Today, surgical operation remains the gold standard in such pathologies, especially mandatory when there is important valvular regurgitation and left-to-right shunt. Currently living in the era of fast and good cosmetic outcomes, minimally invasive and endovascular approaches should be developed and more often practiced. This scientific presentation is the first step in showing our department steps in performing minimally invasive surgeries as a routine.

## 1. Introduction

The atrial septal defect is, following the bicuspid aortic valve, the most common congenital cardiac disease discovered in adults. Ostium secundum-type defects are encountered in about 70% of patients, followed by ostium primum defects, which are commonly associated with malformations of the atrioventricular valves [1]. The association of a cleft anterior mitral valve (left atrioventricular valve) and a primum-type defect is what defines the partial form of an atrioventricular canal. Atrioventricular valve regurgitation through the cleft (or zone of apposition) is also commonly found [2,3].

The diagnosis of this congenital pathology is usually established on routine echocardiography, as symptoms commonly appear after several years or decades of left AV valve regurgitation [4,5,6]. Dyspnea, fatigue, and recurrent respiratory tract infections are the most common clinical symptoms [7,8,9].

The surgical intervention remains the gold standard treatment and it becomes mandatory in the presence of an important left-to-right shunt, cardiomegaly, or significant valvular regurgitation [10,11]. Apart from the traditional approach through midline sternotomy, this lesion is also amenable for correction through a minimally invasive approach, i.e., a right-sided mini-thoracotomy with peripheral cannulation; this alternative technique allows a speedier recovery and better cosmetic results [12,13].

## 2. Case Report

We describe the case of a 20-year-old woman who presented in our department due to a sudden start of symptomatology (shortness of breath) and palpitations on effort. The physical examination was unremarkable. No murmurs were clearly heard. The patient was thin for her height, had no skin modifications, 98% blood oxygen saturation, and normal blood pressure. Jugular venous pressure was normal. There were no signs of decompensated heart failure of peripheral edema.

The EKG showed sinus rhythm, with a right axis deviation, slightly prolonged PR interval, and a right bundle block aspect. The chest X-ray described increased pulmonary circulation, with no acute lesions and no cardiomegaly.

**Figure 1:**
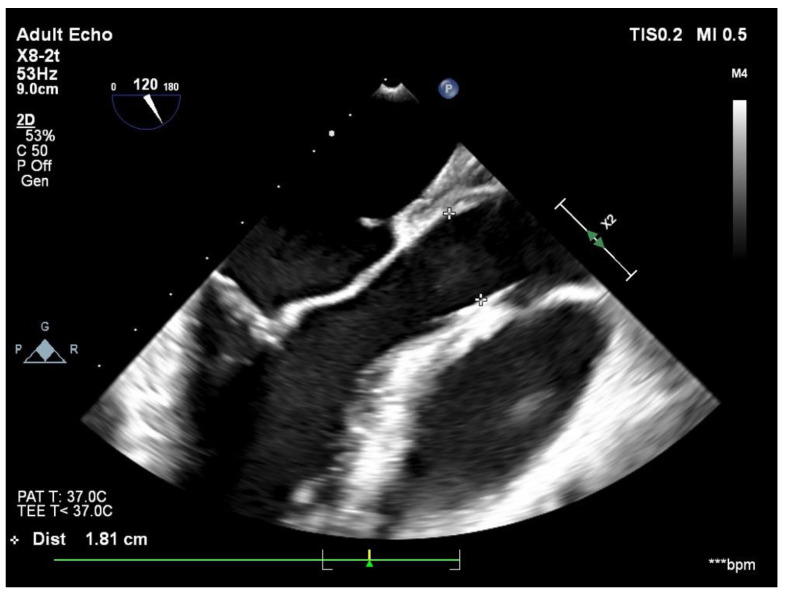
Transesophageal echocardiographic preoperative representation.

The diagnosis was made mainly on transthoracic echocardiography. It revealed that the interatrial septum presented an ostium primum-type defect of around 2 cm and a left-to-right shunt, with a cleft anterior mitral valve and significant mitral regurgitation. In order to better assess the anatomy and the function of the mitral valve, the patient underwent transesophageal echocardiography, which confirmed the ostium primum atrial defect and the severe left AV valve regurgitation that is secondary to the left AV valve cleft (Figure 1). A computer tomography (CT) with contrast substance was performed in order to evaluate the pulmonary venous return and the relation between the thoracic wall and the aorta and the cardiac mass (useful in planning a surgical thoracotomy approach). The CT showed normal pulmonary vein connection and no other significant pathologic modifications.

A diagnosis of partial AVSD with severe regurgitation of the left atrioventricular valve was retained. Given the cosmetic concerns of the patient and considering her young age, a minimally invasive approach was preferred.

**Figure 2:**
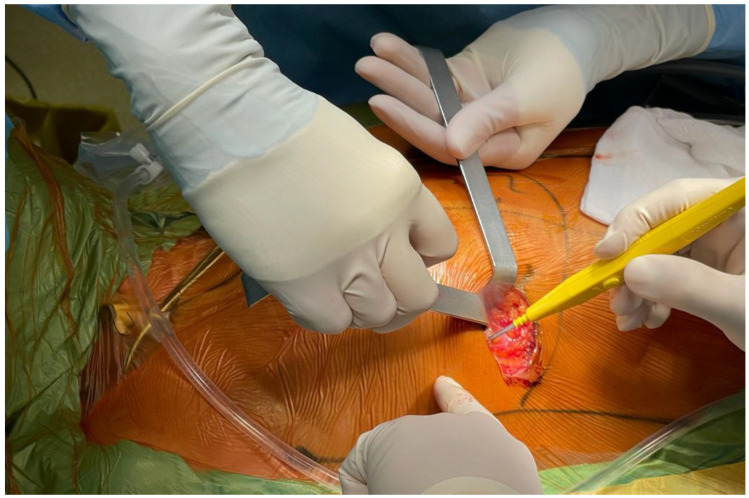
Incision at the level of the 4th intercostal space.

A 5–6 cm skin incision was performed in the right inframammary fold (Figure 2); the chest was entered through the fourth intercostal space. The pericardium was opened 2 cm anterior to the phrenic nerve, and a patch of the autologous pericardium was harvested and preserved in a saline solution. Cardiopulmonary bypass was started using percutaneous femoral and jugular venous and femoral arterial cannulation and mild systemic hypothermia (34 °C). The aorta was cross-clamped through a separate incision and antegrade cardioplegia (Custodiol-Bretschneider) was administered.

A right atriotomy was performed; the OP ASD was large, measuring about 3/2 cm; the edges of the zone of apposition of the left AV valve were slightly thickened. There was no communication at the interventricular level, as the AV valve was attached to the crest of the interventricular septum.

**Figure 3:**
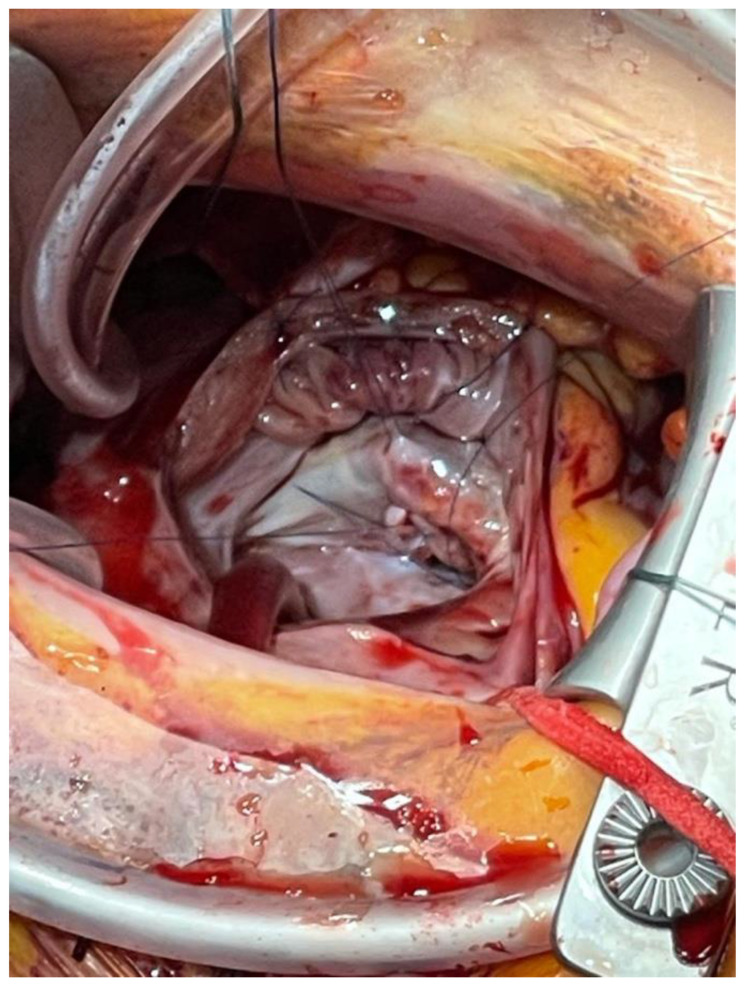
Anterior cleft repair of the mitral valve.

The cleft was closed using multiple “figure-of-eight” polypropylene; the water test showed a competent valve (Figure 3). The orifice of the valve was probed with an adequately sized (no. 27) Hegar dilator and showed no signs of stenosis.

**Figure 4:**
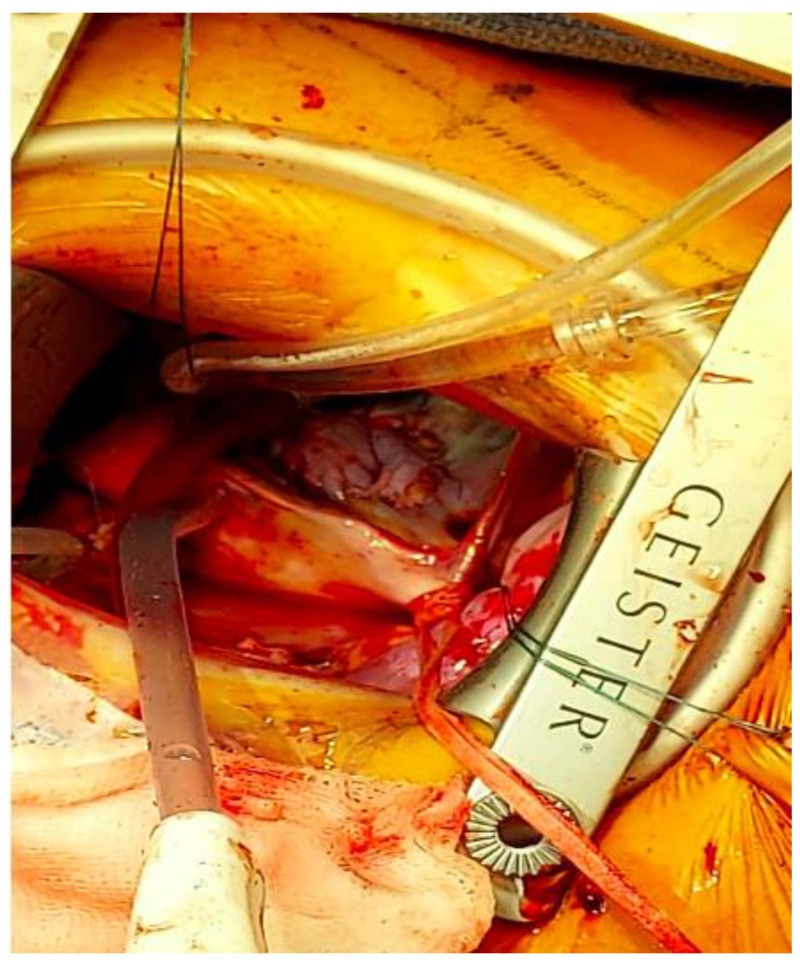
Closure of defect with pericardial patch.

**Figure 5:**
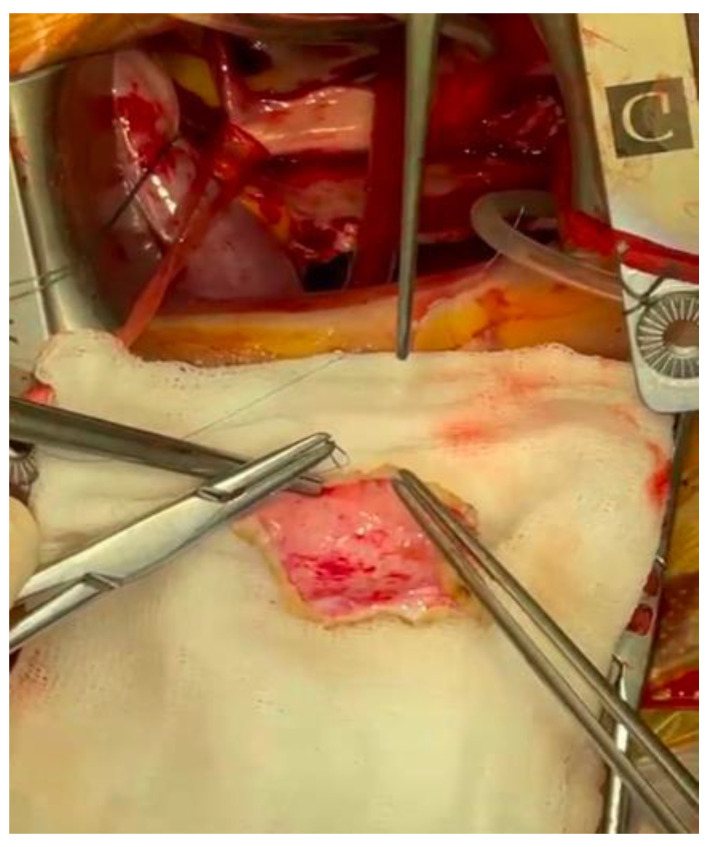
Sizing of pericardial patch.

The defect was closed with the previously harvested pericardial patch, using a continuous polypropylene suture (Figure 4 and Figure 5); the coronary sinus was drained into the right atrium. There was no need for intervention on the right AV valve.

The right atrium was then closed with a running suture. Temporary epicardial pacing wires were fixed to the RV and the aortic clamp was removed. The sinus rhythm resumed spontaneously, and CPB was weaned under small doses of vasopressor support.

Intraoperative transesophageal echocardiography showed no residual shunt, a trivial left AV valve regurgitation and mild right AV valve regurgitation, good biventricular systolic function, and no signs of pulmonary hypertension.

**Figure 6:**
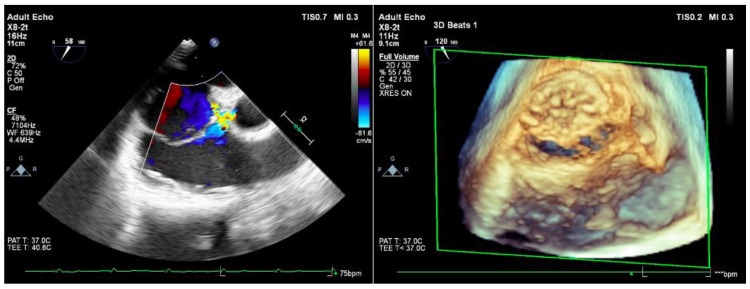
Postoperative transesophageal echocardiographic aspect.

The patient was extubated on the evening of surgery under stable hemodynamics and without any neurological complications. The aspect of the postoperative transesophageal echocardiography is presented in Figure 6. The patient developed a pneumothorax after the removal of the chest drains, which required the percutaneous insertion of a draining tube in the pleural space. After draining the pneumothorax, the tube was removed. There were no complications at the sites of peripheral cannulation. The patient had a very good recovery and was discharged on the seventh postoperative day. The discharge echocardiogram showed a trivial left AV valve regurgitation with no stenosis, mild right AV valve regurgitation, good biventricular contractility, and no residual shunts. Minimal medical treatment was recommended with bisoprolol 2.5 mg twice a day if the pulse rate is higher than 80 bpm. The patient was encouraged to return to a normal healthy lifestyle. She is scheduled to return for a regular routine follow-up with our cardiologist.

## 3. Discussions

This case presentation aims to demonstrate that, in experienced centers, patients with right-sided heart lesions, including ASDs and partial AVSDs, can be repaired using a minimally invasive surgical approach [14,15].

Atrial septal defects have traditionally been surgically repaired through a median sternotomy incision [16,17]. A minimally invasive surgical approach via anterolateral right mini thoracotomy has been associated with a shorter hospitalization period and faster recovery, less blood loss, and better cosmetic results, especially in young patients [18,19]. The safety of the minimally invasive approach has also been established, as the rate of conversion to sternotomy is low, as is the rate of major complications [20].

The aesthetic implications of this approach must be emphasized. The presence of an easily concealable scar reduces the psychological stress associated with operated heart disease. For this reason, there is a worldwide trend in performing more congenital procedures via thoracotomy, in children as well as in adults [13,21,22]. One difference between the two groups is that, in children, central cannulation is easier, due to the short distance between the chest wall and the great vessels. This spares the need for supplemental groin incisions, at the cost of a slightly larger chest incision, required in order to easily access a wider area of the mediastinum. In adults, in order to keep the chest incision small, peripheral cannulation is often required; however, with the advancement of percutaneous cannulation and vessel sealing solutions, the patients will remain with only puncture wounds instead of overt surgical scars.

Most right-sided congenital lesions (ostium secundum, sinus venosus, partial AVSD, some types of VSDs) are amenable to repair via a thoracotomy approach. Most patients thus present favorable anatomy for this approach, except for those cases where peripheral cannulation is difficult due to vascular lesions (such as aortoiliac calcifications or femoro-iliac deep vein thrombosis) or when there are pleural adhesions due to previous pleural effusions or chest wall radiation therapy [23]. Chest wall deformities and extreme patient sizes also represent a challenge for this kind of access and must be judged on a case-by-case basis.

Common complications after the repair of partial AVSDs are residual left or right AV valve regurgitation and arrhythmias, of which complete heart block and sinus node dysfunction are the most frequent [8,24,25,26,27,28]. Residual regurgitation occurs in the setting of a severely dystrophic valve, or when a repair is performed in an adult who has annular dilation (secondary to left ventricular dilation after years of severe left AV valve incompetence) or thickened leaflet edges. In this situation, supplemental procedures might be necessary, such as ring annuloplasty, commissure annuloplasty, or cusp extension [29]. Fortunately, we have been able to cure the left AV valve regurgitation only by closing the zone of apposition, even though the cusp edges were relatively pliable.

Complete heart block occurs due to injury of the AV node and the His bundle, which are located in the proximity of the suture line for the ASD patch (between the coronary sinus and the tricuspid annulus). Some authors prefer to divert this suture line away from the coronary sinus, thus draining it into the left atrium [29]; although of no great clinical significance, it does lead to a loss of about 3–4 points of peripheral saturation, due to the right-left shunt of the coronary sinus. In our case, the patch has been sutured so as to leave the coronary sinus draining to the right atrium, without any postoperative conduction issues.

It was believed that, despite the complexity of the lesion, the repair of a partial atrioventricular canal is associated with low morbidity and mortality even in patients older than 40 years [3,30]. Recent studies have also proved that delayed repair until the third decade of life is frequently associated with a decrease in life expectancy [10,31,32].

## 4. Conclusions

We hereby presented a case of a young 20-year-old patient that was successfully treated in our department for the partial atrioventricular canal. We remind that surgical intervention remains the gold standard treatment and it becomes mandatory in the presence of an important left-to-right shunt, cardiomegaly, or significant valvular regurgitation. In our case, a minimally invasive surgical approach was used, having as results a shorter hospitalization with a speedy recovery and better cosmetic results for the patient. The patient was discharged less than 7 days after surgery. Furthermore, 6 months after the intervention, the patient returned for a routine follow-up, and on ETT, the patient presented no complications, with a favorable evolution. In our clinical department, such cases become routine, practiced by our young team of cardiac surgeons. By this means, minimally invasive surgical approaches grow and become the gold standard.

## Data Availability

The data presented in this study are available on reasonable request from the corresponding author.
